# Pregnancy and delivery after surgical remission in women with prolactinomas desiring pregnancy: Refining surgical indications

**DOI:** 10.1007/s11102-026-01648-y

**Published:** 2026-03-25

**Authors:** Kosaku Amano, Yasufumi Seki, Yuichi Oda, Shihori Kimura, Kaoru Yamashita, Atsuhiro Ichihara, Takakazu Kawamata

**Affiliations:** 1 Department of Neurosurgery, Tokyo Women’s Medical University, 8-1 Kawada-Cho, Shinjuku-Ku, Tokyo, 162-8666 Japan; 2 Department of Medicine, Division of Hormonal Medicine and Bioregulatory Science, Tokyo Women’s Medical University, 8-1 Kawada-Cho, Shinjuku-Ku, Tokyo, 162-8666 Japan

**Keywords:** Prolactinoma, Surgical remission, Pregnancy, Potential cure, Transsphenoidal surgery, Cabergoline

## Abstract

**Objective:**

Prolactinomas are usually managed with cabergoline, and surgery is reserved for strictly selected cases. Based on our surgical experience, we observed no recurrence in women who conceived and delivered after surgical remission. We therefore evaluated surgical outcomes and indications in pregnancy-seeking women.

**Methods:**

We retrospectively reviewed 57 women who underwent surgery in 1998–2008 (median age 28.0 years) and identified predictors of remission and recurrence. Refined criteria (desiring pregnancy, enclosed-type tumor, and Knosp grade ≤ 2 with anticipated complete resection) were then applied to 135 women treated in 2009–2022 (median age 35.0 years).

**Results:**

In 1998–2008, remission was achieved in 73.8% of enclosed-type microadenomas and improved to 95.2% in the later period. Postoperative prolactin < 3 ng/mL predicted lower recurrence than 3–15 ng/mL (4.3% vs 50.0%, *P* = 0.0098). In 2009–2022, 25/135 (18.5%) underwent surgery. Of 32 women who met the refined criteria, 20 elected surgery, and all achieved remission without new deficits. Across both cohorts, 59 women of childbearing age achieved remission. Recurrence occurred in 6 of 30 women who did not conceive (20%) but in none of the 29 who conceived and delivered (0%, *P* = 0.0237).

**Conclusions:**

Pregnancy and delivery following surgical remission were associated with no recurrence, suggesting a potential cure in this subgroup. When performed by experienced pituitary surgeons, transsphenoidal surgery may offer a curative pathway for carefully selected women desiring pregnancy. These findings support further refinement of surgical candidacy in pregnancy-seeking women and warrant validation in larger cohorts.

## Introduction

Prolactin-producing pituitary adenomas (prolactinomas) represent the most common subtype of functional pituitary adenomas, accounting for approximately 40% of all cases [1]. They predominantly affect women of reproductive age and are frequently associated with menstrual irregularities, amenorrhea, galactorrhea, and infertility. When tumors enlarge substantially, visual disturbances may also occur. Given this demographic and symptom profile, the management of prolactinomas in women, particularly those desiring fertility, presents a unique clinical challenge.

Dopamine agonists (DAs) remain the first-line treatment for prolactinomas. Cabergoline (CAB), a long-acting DA, has demonstrated high efficacy in normalizing prolactin levels and inducing tumor shrinkage with relatively few side effects; accordingly, it is widely recommended as initial therapy for most patients [1–10]. Surgery has traditionally been reserved for DA resistance/intolerance or complications such as cerebrospinal fluid (CSF) leakage, but is now also recognized as an alternative option in carefully selected patients.

However, in women desiring pregnancy, long-term DA therapy poses unique limitations. CAB is usually stopped after pregnancy is confirmed; although tumor enlargement is uncommon in microprolactinomas, closer monitoring is warranted in selected patients with larger or residual tumors, and many require postpartum reassessment of DA therapy. Additionally, many patients require long-term DA therapy to maintain biochemical control.

On the other hand, in our clinical practice, we observed that women who achieved surgical remission and subsequently conceived and delivered experienced no recurrence, whereas recurrence occurred among women who did not conceive after surgical remission. This observation led us to refine surgical indications in women of childbearing age with tumors assured of complete resection. The present study investigates surgical outcomes under these criteria and discusses the potential of transsphenoidal surgery (TSS) as a rational primary treatment option in women desiring pregnancy.

## Methods

We retrospectively reviewed 57 female patients aged 20 to 61 years (mean: 30.3, median: 28.0) who underwent surgery for pathologically confirmed prolactinomas at Tokyo Women’s Medical University Hospital between 1998 and 2008. Of these, 56 procedures were performed via the transsphenoidal approach and one via open craniotomy. Surgical outcomes—including remission, recurrence, and pregnancy following remission—were analyzed.

Based on these findings, we refined surgical indications for women of childbearing age, specifically those desiring pregnancy and presenting with enclosed-type prolactinomas considered amenable to complete resection with Knosp grade ≤ 2. These criteria were prospectively applied to a subsequent cohort of 135 female patients aged 13 to 77 years (mean: 34.0, median: 35.0) treated between 2009 and 2022, in order to evaluate the appropriateness of these indications. In reproductive-age women, treatment decisions were patient-driven following multidisciplinary counseling, with endocrinologists outlining the benefits and limitations of dopamine agonist therapy, and neurosurgeons explaining the surgical approach, its potential advantages, and possible complications.

Serum prolactin (PRL) levels were measured using the radioimmunoassay (RIA) kit (Eiken Chemical Co., Tokyo, Japan) until January 2002, the electrochemiluminescence immunoassay (ECLIA) kit (Tosoh, Tokyo, Japan) until 2012, and since April 2012 the Elecsys Prolactin III assay (Roche Diagnostics, Tokyo, Japan), which is standardized to the Third International Reference Preparation (WHO Reference Standard-PRL, 84/500). Normal serum PRL ranges were defined as 4.91–29.32 ng/mL for premenopausal women, and 3.12–15.39 ng/mL for postmenopausal women. Surgical remission was defined as a postoperative PRL concentration < 15 ng/mL. Recurrence was defined as two consecutive measurements exceeding 30 ng/mL after remission.

All patients or their guardians provided informed consent. The study was approved by the Ethics Committee of Tokyo Women’s Medical University (approval number: 2021–0063) and conducted in accordance with the Declaration of Helsinki and the STROBE (Strengthening the Reporting of Observational Studies in Epidemiology) guidelines.

Categorical variables (e.g., remission, recurrence, pregnancy, delivery) were compared using Fisher’s exact test, while continuous variables (e.g., time to recurrence) were summarized descriptively. A two-sided *p*-value < 0.05 was considered statistically significant. Statistical analyses were performed using Python (version 3.11; Python Software Foundation) with the SciPy (version 1.11) and statsmodels (version 0.14) libraries.

## Results

### Surgical indications and outcomes during the early period (1998–2008)

A total of 57 female patients aged 20 to 61 years (mean: 30.3, median: 28.0) underwent surgery for prolactinomas between 1998 and 2008. Surgical indications included 42 patients (73.7%) with enclosed-type microadenomas who selected surgery by patient preference (remission rate, 73.8%), 9 (15.8%) with dopamine agonist (DA) resistance or intolerance (44.4%), 5 (8.8%) with suspected stalk effect (20.0%), and 1 (1.8%) with poor medical adherence (0%).

Among 31 patients with enclosed-type microadenomas who achieved remission by patient choice, the remission rate improved markedly from 52.4% (11/21) in the first half of the study period (1998–July 2003) to 95.2% (20/21) in the latter half (August 2003–2008) (*P* < 0.001). Serum PRL levels on postoperative days (POD) 1–10 were predictive of recurrence: recurrence occurred in 1 of 23 patients (4.3%) with postoperative PRL < 3 ng/mL, compared with 4 of 8 patients (50%) with PRL between 3–15 ng/mL (*P* = 0.005) (Table 1). Overall, 42/57 patients were operated for enclosed-type microadenomas with curative intent, whereas the remaining 15 underwent surgery for other indications (DA resistance/intolerance, suspected stalk effect/non-prolactinoma, or poor compliance), in which anticipated gross-total resection was not always the primary objective. Remission in this non–curative-intent subgroup was correspondingly lower (5/15, 33.3%). Given the heterogeneity of tumor size and invasiveness in these 15 patients, detailed imaging variables for the entire early cohort were not tabulated in Table 1.

**Table 1:** Surgical indications and outcomes of 57 women with prolactinomas (1998–2008)

Age (range/mean/median), years	20–61/30.3/28.0		
	*n* (%)	Remission, *n* (%)	*p*-value
Surgical indication	57	36 (63.2)	
Enclosed-type microprolactinoma (patient preference)	42 (73.7)	31/42 (73.8)	
- Former vs Latter period		11/21 (52.4) vs 20/21 (95.2)	0.0036
Resistance or intolerance to medical therapy	9 (15.8)	4/9 (44.4)	
Preoperative diagnosis of non-prolactinoma	5 (8.8)	1/5 (20.0)	
Poor compliance	1(1.8)	0/1 (0)	
Among remission cases of Enclosed-type microprolactinoma (*n* = 31)		Recurrence, *n* (%)	
Serum prolactin (POD 1–10), ng/mL Overall		5/31 (16.1)	
< 3 vs 3–15		1/23 (4.3) vs 4/8 (50.0)	0.0098

Former period: 1998–July 2003; latter period: August 2003–2008, POD: postoperative day

Remission: postoperative normoprolactinemia without radiological evidence of residual tumor (see Methods)

Recurrence: two consecutive serum prolactin measurements > 30 ng/mL during follow-up (see Methods)

### Surgical indications and outcomes under refined criteria (2009–2022)

Between 2009 and 2022, 135 female patients aged 13 to 77 years (mean: 34.0, median: 35.0) were treated. Of these, 115 patients (85.2%) initially received CAB. CAB monotherapy was successful in 110 patients (81.5%), while 5 patients (3.7%) required surgery due to CAB resistance.

**Fig. 1:**
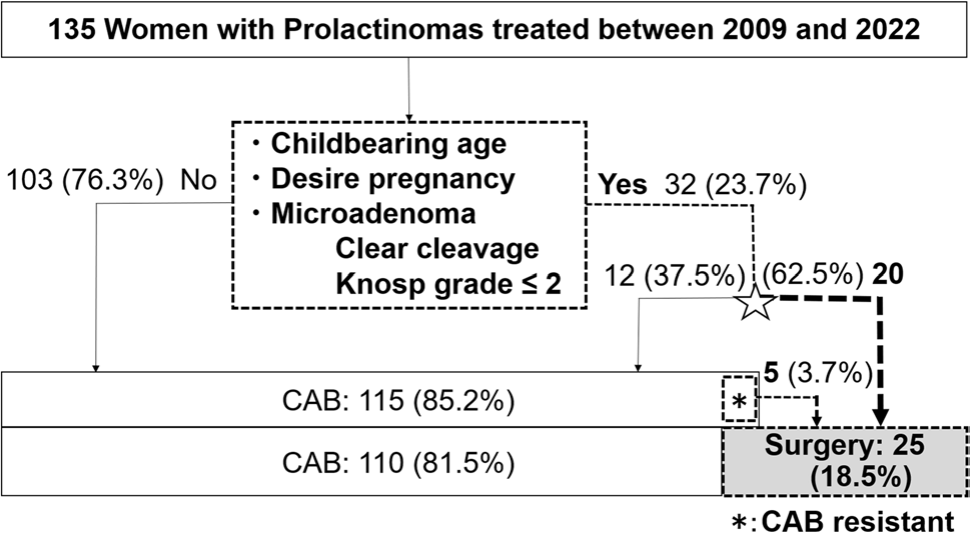
Treatment flowchart of 135 female patients with prolactin-producing pituitary adenomas treated between 2009 and 2022

In total, 25 patients (18.5%) underwent surgical treatment. Surgical indications included 20 patients (80%) with enclosed-type microadenomas (Knosp grade ≤ 2, with assured complete resection) who elected surgery by personal preference (remission rate, 100%), and 5 (20%) with CAB resistance or intolerance (remission rate, 60%). Among 32 women of childbearing age who met the refined surgical criteria—defined as enclosed-type tumor, desire for pregnancy, and Knosp grade ≤ 2—20 (62.5%) opted for surgery, and all achieved surgical remission (Fig. 1; Tables 2 and 3). The remaining five of the 25 surgically treated patients did not meet the refined pregnancy-seeking criteria because surgery was performed for CAB resistance or intolerance (Cases 21–25; Table 3; Fig. 1*). Among these five patients, the two without biochemical remission had macroprolactinomas, whereas the three with POD1–10 PRL < 3 ng/mL had microprolactinomas.

**Table 2:** Treatment overview of women with prolactinomas (2009–2022)

	Overall	CAB therapy; *n* (%)	Surgery; *n* (%)
*n*	135	110 (81.5)	25 (18.5)
Age (range/mean/median), years	13–77/34.0/35.0	13–77/34.1/34.5	22–67/33.5/35.0
Surgical indication (among surgical cases)		*n* = 25 (%)	Remission, *n* (%)
Enclosed-type microprolactinoma (patient preference)		20 (80)	20 (100)
Resistance or intolerance to CAB		5 (20)	3 (60)

*CAB* cabergoline

Remission: postoperative normoprolactinemia without radiological evidence of residual tumor (see Methods)

**Table 3:** Clinical characteristics and outcomes of surgically treated women with prolactinomas (2009–2022)

Case	Age	Desire	Married	Postoperative PRL	Pregnancy		Adjuvant treatment
No		pregnancy	or with fiancé	in POD 1–10*	and delivery	Recurrence	after surgery
Childbearing women with clear cleavage and Knosp ≤ 2 tumor who desired pregnancy and chose surgery							
1	25	Yes	No	< 3	No	No	
2	22	Yes	Yes	< 3	Yes	No	
3	36	Yes	No	< 3	No	No	
4	35	Yes	No	< 3	Yes	No	
5	36	Yes	No	3–15	No	Yes	CAB after recurrence
6	22	Yes	Yes	< 3	No	No	
7	35	Yes	No	< 3	No	No	
8	40	Yes	Yes	< 3	No	No	
9	26	Yes	Yes	< 3	Yes	No	
10	35	Yes	Yes	< 3	Yes	No	
11	38	Yes	Yes	3–15	Yes	No	
12	36	Yes	No	< 3	No	No	
13	38	Yes	Yes	< 3	Yes	No	
14	34	Yes	Yes	< 3	Yes	No	
15	29	Yes	Yes	3–15	Yes	No	
16	43	Yes	No	< 3	No	No	
17	35	Yes	No	< 3	No	No	
18	27	Yes	Yes	3–15	Yes	No	
19	28	Yes	Yes	3–15	Yes	No	
20	30	Yes	Yes	< 3	Yes	No	
CAB-resistant cases							
21	31	No	No	No remission	No	Not applicable	GK
22	64	No	Yes	No remission	No	Not applicable	Surgeries, GK, TMZ
23	38	Yes	Yes	< 3	No	No	
24	31	Yes	No	< 3	Yes	No	
25	24	Yes	No	< 3	Yes	No	

^*^ PRL category: < 3 = < 3 ng/mL; 3–15 = 3–15 ng/mL.; TMZ, Temozolomide; CAB, Cabergoline; GK, Gamma knife

Remission: postoperative normoprolactinemia without radiological evidence of residual tumor (see Methods)

Recurrence: two consecutive serum prolactin measurements > 30 ng/mL during follow-up (see Methods)

Conventional indications (CAB resistance/intolerance) accounted for 5 of 25 surgical cases (20.0%) and 3.7% of the overall cohort (5/135). The inclusion of pregnancy-seeking, curative-intent surgery as an additional indication contributed to a total surgical rate of 18.5% (25/135) in this cohort (Fig. 1).

### Reproductive outcomes and recurrence in women of childbearing age following surgical remission

A total of 59 women of childbearing age (range, 20–43; mean, 29.4; median, 29.0 years) achieved surgical remission between 1998 and 2022. Surgical indications included 52 enclosed-type microadenomas by patient preference, 4 with DA resistance, and 3 with intolerance. Postoperative follow-up duration was calculated from the date of surgery to the last clinical assessment (censoring date: December 31, 2025). Median follow-up was 16.7 years (IQR, 11.3–20.0; range, 3.1–26.7) in women who conceived and delivered after remission, and 19.3 years (IQR, 17.1–22.5; range, 7.5–27.5) in women who did not conceive. Follow-up was longer in the non-conception group (Mann–Whitney U test, two-sided *p* = 0.0296). The overall recurrence rate was 10.2% (6/59); 13.9% (5/36) in 1998–2008 and 4.3% (1/23) in 2009–2022, with no significant difference between periods (*P* = 0.430).

Time to recurrence among the 6 recurrent cases ranged from 2 years 5 months to 6 years 7 months, with a median of 4 years 9 months (IQR, interquartile range, 2 years 10 months to 6 years 6 months). Twenty-nine women (49.2%) conceived and delivered after surgery. The median interval from surgery to estimated conception—defined for descriptive purposes as 40 weeks (280 days) before the delivery date—was 2 years 3 months (IQR, 9 months to 3 years 11 months; range, 2 months to 17 years 8 months). No recurrences were observed in women who conceived and delivered (0/29, 0%), whereas recurrence occurred in 6 of 30 women who did not conceive (20%) (*P* = 0.0237). The recurrence rate among non-pregnant women was 25% (5/20) in 1998–2008 and 10% (1/10) in 2009–2022. While subgroup comparisons within each period were not statistically significant (*P* = 0.527 and *P* = 0.435, respectively), the overall difference between the pregnant and non-pregnant groups was statistically significant (*P* = 0.0237). Preoperative serum PRL levels did not differ between women who conceived and delivered after surgical remission and those who did not (median [IQR], 117.1 [91.7–208.9] vs 130.8 [97.4–162.3] ng/mL; range, 52.3–716.3 vs 39.1–339.0; Mann–Whitney U test, two-sided *p* = 0.994). Among women without pregnancy/delivery, preoperative serum PRL levels also did not differ between recurrent and nonrecurrent cases (median [IQR], 132.6 [86.2–166.6] vs 130.8 [98.5–156.3] ng/mL; Mann–Whitney U test, two-sided *p* = 0.86). The Ki-67 labeling index also did not differ between women who conceived and delivered after surgical remission and those who did not (median [IQR], 1.4 [0.7–2.1] vs 1.25 [0.7–2.45]; range, 0.3–6.0 vs 0.5–6.3; Mann–Whitney U test, two-sided *p* = 0.914). Among women without pregnancy/delivery, recurrent cases tended to have a higher Ki-67 index than nonrecurrent cases, although this did not reach statistical significance (median [IQR], 2.75 [2.05–3.5] vs 0.8 [0.7–1.6]; Mann–Whitney U test, two-sided *p* = 0.094). Postoperative gonadal function was preserved, and no new pituitary hormonal deficits were identified on postoperative dynamic endocrine testing, making differential postoperative gonadal dysfunction an unlikely explanation for the observed recurrence-free association.

The proportion of women who conceived and delivered after surgical remission increased from 44.4% (16/36) in the early period (1998–2008) to 56.5% (13/23) in the later period (2009–2022), although this difference did not reach statistical significance (Fisher’s exact test, two-sided: p = 0.430; OR = 1.63, 95% CI 0.57–4.66). (Tables 3 and 4).

**Table 4:** Reproductive outcomes and recurrence in childbearing women with prolactinomas following surgical remission

	Overall	1998–2008	2009–2022	*p*-value †
*n*	59	36	23	
Age (range/mean/median), years	20–43/29.4/29.0	20–36/27.5/26.0	22–43/32.7/35.0	
Preoperative serum prolactin level (range/median), ng/mL	40–716/127.7	40–716/116.8	64–370/131.2	
Surgical indication				
Enclosed-type microprolactinoma (patient preference)	52	32	20	
Resistance to dopamine agonists	4	1	3	
Intolerance to dopamine agonists	3	3	0	
Postoperative outcomes				
Pregnancy and delivery	29 (49.2%)	16 (44.4%)	13 (56.5%)	0.430
No pregnancy and delivery	30 (50.8%)	20 (55.6%)	10 (43.5%)	0.430
Recurrence	6 (10.2%)	5 (13.9%)	1 (4.3%)	0.389
- Recurrence among women with pregnancy and delivery	0/29 (0%)	0/16 (0%)	0/13 (0%)	
- Recurrence among women without pregnancy and delivery	6/30 (20%)	5/20 (25%)	1/10 (10%)	0.633
- *p*-value (pregnancy and delivery vs no pregnancy and delivery)	0.0237	0.527	0.435	

^†^ Periods: 1998–2008 vs 2009–2022

Remission: postoperative normoprolactinemia without radiological evidence of residual tumor (see Methods)

Recurrence: two consecutive serum prolactin measurements > 30 ng/mL during follow-up (see Methods)

**Fig. 2:**
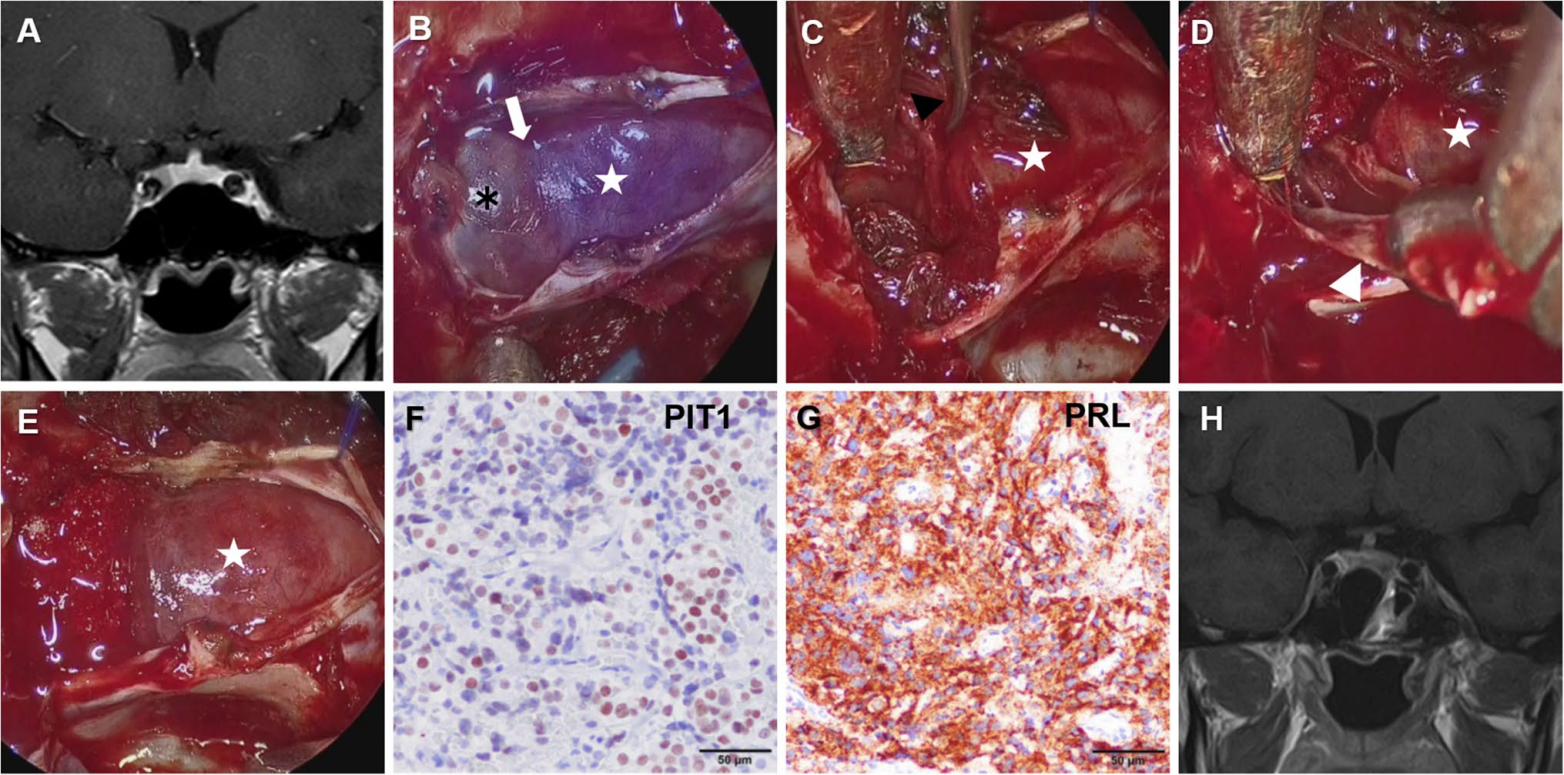
Representative case of an enclosed-type prolactinoma with surgical remission (Table 3, Case No, 19). **A **Preoperative contrast-enhanced coronal T1-weighted MRI showing a well-demarcated prolactinoma located on the right side with Knosp grade 2 invasion. **B** Intraoperative indocyanine green fluorescence (ICG) endoscopic view. The normal pituitary gland (star) is fluorescent under ICG, the tumor is indicated by an asterisk, and the arrow denotes the cleavage plane between tumor and gland. **C** Endoscopic view showing dissection of the pseudocapsule (black arrowhead) from the normal pituitary gland (star). **D** Endoscopic view during removal of the inner wall of the cavernous sinus infiltrated by tumor (white arrowhead), with the normal pituitary gland (star). **E** Endoscopic operative field after complete tumor removal, with the preserved pituitary gland (star) and the tumor cavity filled with collagen sponge. **F** Immunohistochemistry for PIT1 confirming pituitary cell lineage. **G** Immunohistochemistry showing strong prolactin (PRL) expression. **H** Postoperative T1-weighted MRI demonstrating complete resection with preserved pituitary gland

A representative case is shown in Fig. 2 (Table 3, Case No. 19). This 28-year-old woman, who was engaged at the time of diagnosis, presented with menstrual irregularities and was found to have a serum PRL level of 208.0 ng/mL. She elected to undergo surgery, which reduced her postoperative PRL to 7.7 ng/mL. Following marriage, she conceived and delivered, and has remained free of recurrence for two years thereafter.

## Discussion

### Current role of Cabergoline and surgical indications

Cabergoline (CAB) remains the gold standard for treating prolactin-producing pituitary adenomas (prolactinomas) due to its high efficacy in normalizing serum prolactin (PRL) levels and reducing tumor volume [1–10], even in the presence of visual impairment [10]. Compared with older dopamine agonists (DAs) such as bromocriptine and terguride, CAB offers milder and fewer side effects [1–10]. In 2010, Klibanski comprehensively outlined surgical indications for prolactinomas, including tumor progression despite optimal medical therapy, pituitary apoplexy, DA intolerance or resistance, fertility-seeking women with DA-resistant microadenomas or chiasmal compression, unresponsive cystic tumors, CSF leakage during DA use, and cases with psychiatric contraindications to DAs [2]. Subsequently, surgical indications for prolactinomas were reported from a neurosurgical perspective [10–18]. Among these, absolute indications have remained limited to CAB resistance, intolerance, and CSF leakage, while relative surgical indications include unresponsive cystic prolactinomas [10, 19–21] and cranial nerve palsy [22]. Other potential reasons for preferring surgery over medical therapy include avoidance of lifelong medication, cost-effectiveness [23, 24], early pregnancy planning, and improved fertility outcomes after surgery [25]. However, these factors remain inconclusive and do not establish surgery as superior. Surgical intervention should be considered only with clear indications and may be offered as a meaningful alternative to medical therapy when it can be performed safely and appropriately.

We retrospectively analyzed surgically treated prolactinoma cases between 1998 and 2008 and found that no recurrence occurred among women who conceived and delivered after achieving surgical remission. Based on this emerging finding, we propose a refined surgical indication for carefully selected pregnancy-seeking women—curative-intent surgery followed by pregnancy and childbirth after surgical remission—as an alternative to long-term dopamine agonist therapy. Since 2009, we have prospectively applied a standardized, magnetic resonance imaging (MRI)-driven candidacy strategy focused on enclosed tumors and minimal cavernous sinus invasion.

### A noble surgical indication for childbearing women desiring pregnancy

Despite its potential clinical significance, neither the 2023 international consensus statement by the Pituitary Society [8] nor recent comprehensive review of prolactinoma treatments [9] addressed the relationship between pregnancy and delivery following surgical remission and recurrence. However, in our current study of 59 childbearing women who achieved remission following surgery between 1998 and 2022, none of the women who became pregnant and delivered experienced recurrence (0%, 0/29), whereas recurrence occurred in 20% (6/30) of those who did not conceive (*P* = 0.0237; Table 4). In our cohort, pregnancy and delivery after surgical remission were associated with an absence of recurrence. However, given the observational design, this association should be interpreted cautiously and may be influenced by selection factors related to surgical candidacy and postoperative biochemical status.

 Although patients can become pregnant while on CAB therapy, continuation is usually required postpartum. Notably, the Pituitary Society guidelines recommend at least two-year period of PRL normalization without residual tumor on MRI before attempting withdrawal [26–28], and the likelihood of discontinuing CAB therapy after remission is not high. However, in our series, patients who became pregnant and delivered after surgical remission did not require CAB therapy postpartum and did not experience recurrence. These outcomes are consistent with the possibility of durable, treatment-free remission in carefully selected pregnancy-seeking women after confirmed complete resection. Rather than establishing superiority over CAB, our data suggest that surgery may represent a clinically meaningful option for selected patients when complete resection is anticipated and surgery can be performed safely. In this context, the potential benefit is durable remission without the need for postpartum CAB therapy.

In addition to the advantage of enabling early pregnancy planning (as soon as one month after surgery) and breastfeeding after delivery, surgery offers the unique benefit of potentially achieving long-term remission. When postoperative normoprolactinemia is achieved without radiological evidence of residual tumor, patients may require only annual serum PRL monitoring. However, surgery necessitates hospitalization, carries risk of complications [29, 30], and may cause psychological distress, including anxiety about surgery. Patients must receive balanced, comprehensive counseling—on CAB therapy from endocrinologists and on surgical options from neurosurgeons. Shared decision-making is essential, as one-sided explanations may result in biased or incomplete understanding. Patients should be fully informed of both options and supported in choosing the most appropriate treatment for their individual circumstances. As a result, 20 of 32 childbearing women (62.5%) with enclosed-type prolactinomas who desired pregnancy elected surgery (Fig. 1 star).

### Surgical strategy and patient selection

The number of surgically treated cases decreased over time (57 procedures in 1998–2008 vs 25 in 2009–2022). This likely reflects the broader adoption of CAB with improved tolerability compared with earlier dopamine agonist practice patterns. In addition, our approach to surgical candidacy evolved after 2009: “patient preference” was previously interpreted more broadly, sometimes including women expressing a non-specific future desire for pregnancy, and candidacy was occasionally determined predominantly within the neurosurgical service. Since 2009, we have applied more stringent, MRI-driven criteria for anticipated complete resection and a realistic near-term pregnancy plan, with multidisciplinary counseling that includes endocrinologists and final treatment selection based on informed patient decision-making.

The surgical goal in women desiring pregnancy is total tumor removal with normalization of PRL. Residual tumor necessitates postoperative CAB therapy, thereby defeating the purpose of surgery. Careful patient selection based on detailed preoperative MRI is therefore essential. Suitable candidates have enclosed tumors, a distinct cleavage plane from the normal gland, and minimal cavernous sinus invasion (Knosp grade ≤ 2) (Fig. 1). We selected Knosp grade ≤ 2 as an eligibility threshold because, in our practice, this range still allows reliable identification of an enclosed tumor with a discernible cleavage plane and a high likelihood of complete resection on preoperative MRI. We acknowledge the ongoing discussion regarding whether stricter criteria (e.g., Knosp ≤ 1) should be used to further maximize the probability of gross-total resection, and that the optimal cutoff may depend on imaging interpretation and surgical expertise. Remission rates for enclosed-type tumors improved from 52.4% (1998–July 2003) to 95.2% (August 2003–2008) (*P* = 0.0036, Table 1) due to technical advances in transsphenoidal surgery (TSS). However, even a 95.2% remission rate was insufficient; for women desiring pregnancy, only 100% remission is acceptable. In our series, MRI-based selection with Knosp ≤ 2 achieved 100% remission in pregnancy-seeking women without new deficits, supporting the practicality of this criterion in experienced hands. Nonetheless, these results should be interpreted cautiously and require external validation across centers with varying surgical volumes and expertise.

To achieve this**,** we refined endoscopic techniques, improved TSS instruments [31–34], and adopted high-definition endoscopy [35]. Furthermore, we aim to avoid over-resection to preserve pituitary function [36], which is essential for conception. PRL levels during postoperative days 1–10 (POD1–10) predicted recurrence: patients with PRL < 3 ng/mL had significantly lower recurrence than those with 3–15 ng/mL (4.3% vs. 50.0%, *P* = 0.0098; Table 1). Although postoperative PRL level < 3 ng/mL is ideal for minimizing recurrence, levels of 3–15 ng/mL were considered acceptable if conception was anticipated, prioritizing function preservation. In our cohort, no new postoperative hypopituitarism was identified on dynamic endocrine testing: the growth hormone, hypothalamic–pituitary–adrenal, gonadal, and posterior pituitary axes were all preserved. Accordingly, no patient required new long-term hormone replacement therapy after surgery. To reconcile these competing goals, we emphasize pseudocapsule resection [36], meticulous identification and preservation of the normal pituitary gland [35], and prevention of complications such as CSF leakage [29, 30, 32]. These procedures require an experienced pituitary neurosurgeon [13, 17]. However, even with expert surgical skill, dynamic MRI is critical for identifying the cleavage plane between the tumor and the gland. Furthermore, when the tumor extensively invades the cavernous sinus (Knosp grade > 3) [22, 37] and complete resection cannot be guaranteed, surgery should not be offered to women desiring pregnancy, even in cases of strong patient preference. Importantly, the clinical decision-making framework for surgical candidacy was not standardized in the earlier period. Before 2009, “patient preference” was interpreted more broadly and documentation of near-term pregnancy intent and MRI-based predictors of complete resection was not uniformly incorporated into the surgical indication process. Since 2009, we have implemented a standardized, MRI-driven selection strategy focused on anticipated complete resection and a realistic near-term pregnancy plan, supported by multidisciplinary counseling and informed patient decision-making. This evolution in candidacy assessment likely contributed to the reduced number of surgical cases over time and to the more consistent outcomes observed in the later cohort. Since 2009, a 100% remission rate has been achieved in childbearing women who met the surgical criteria without new hormonal deficits or complications, as a result of meticulous preoperative MRI-based case selection and advances in transsphenoidal surgical instruments and techniques. Although the difference was not statistically significant, the recurrence rate was lower in 2009–2022 than in 1998–2008 (1/10 [10%] vs. 5/20 [20%], *P* = 0.633). This trend may reflect refined patient selection and the higher proportion of women who conceived and delivered (44.4% [16/36] vs 56.5% [13/23], *P* = 0.430; Table 4). This increase may, in part, be related to strategic refinements intended to minimize ascertainment bias when assessing a woman’s intent to pursue pregnancy. If childbearing women are simply asked whether they wish to have a baby, most will naturally respond affirmatively; however, not all will be able to conceive after surgical remission due to various personal or social circumstances. Therefore, to better identify candidates for surgery with curative intent, additional factors—such as marital status, the presence of a partner, and concrete plans for conception—should be assessed preoperatively. In light of recent societal trends such as delayed marriage and childbearing and shifting attitudes toward marriage and child-rearing, we implemented a strategy that included confirming marital status or the presence of a fiancé/stable partner and requiring a commitment to conceive within two years of surgery. This 2-year benchmark was chosen based on our observation that recurrence, when it occurred, did not present earlier than two years postoperatively. In 2009–2022, recurrence occurred in only one woman who did not conceive, at 2.8 years after surgery. The proportion of women who did not become pregnant and deliver decreased from 55.6% (20/36) to 43.5% (10/23), although this difference did not reach statistical significance (*P* = 0.430; Table 4). This decrease may be related to the implementation of stricter selection criteria.

## Hypothesis, limitations, and future directions

The reason why women with prolactinomas who became pregnant and delivered after surgical remission did not experience recurrence remains biologically unexplained. Although the following discussion is speculative, it may provide a possible biological explanation and a framework for future investigation. A few studies have suggested that pregnancy may contribute to sustained remission in prolactinoma [38–40]. One plausible explanation is that pregnancy and childbirth may induce long-lasting neuroendocrine changes that support long-term disease control. Specifically, dopaminergic receptor remodeling during pregnancy may play a key role. Several studies have shown that the hypothalamic–pituitary axis undergoes adaptive changes during gestation, including upregulation of dopamine D2 receptor density in the anterior pituitary and enhanced dopaminergic tone as a compensatory response to hyperestrogenemia [41–43]. These changes may result in prolonged suppression of prolactin secretion, even in the absence of pharmacologic intervention. Following delivery, the re-establishment of dopaminergic feedback—possibly facilitated by a neuroendocrine reset or pregnancy-induced immune tolerance—may create a biochemical environment that is unfavorable for tumor regrowth. These adaptations may be particularly effective following complete tumor resection, which eliminates residual adenoma cells that might otherwise evade neuroendocrine regulation.

We hypothesize that pregnancy may trigger durable neuroendocrine remodeling of the hypothalamic–pituitary axis, involving restoration of dopamine receptor sensitivity, stabilization of prolactin feedback mechanisms, and reprogramming of hormonal homeostasis. Surgical remission may provide a favorable baseline condition for these pregnancy-induced changes to proceed without interference from residual tumor tissue. This hypothesis warrants further investigation using molecular, imaging, or animal models to elucidate the biological basis for long-term disease control or potential cure following pregnancy.

A major limitation of our study is the relatively small number of patients (*n* = 29) who became pregnant and delivered following surgical remission. Nonetheless, the consistency of the findings across two independent cohorts, the complete absence of recurrence, represents clinically meaningful and striking. However, they do not establish causality. Confounding factors such as overall health status, fertility potential, hormonal milieu, or even psychosocial differences may underlie the observed association between pregnancy and sustained remission. Among the 29 women who conceived and delivered after surgical remission, 4 achieved pregnancy following fertility treatment. This observation suggests that the association with sustained remission may relate primarily to pregnancy and delivery themselves rather than to the mode of conception. Notably, the pregnancy group also included three women with CAB-resistant prolactinomas, none of whom experienced recurrence, suggesting that the association was not limited to CAB-responsive tumors. If the molecular and hormonal mechanisms responsible for this non-relapsing state can be identified, they may serve as novel therapeutic targets. Medical strategies that replicate the endocrine milieu induced by surgery followed by pregnancy could potentially induce long-term remission without surgical intervention or allow for safe discontinuation of dopamine agonists. These insights provide not only a rationale for re-evaluating surgical indications in selected patients but also a novel biological framework for developing curative strategies for prolactinomas.

Given the observational nature of this study, the absence of recurrence in the pregnancy group must be interpreted cautiously. Nonetheless, it provides a valuable hypothesis-generating insight into a possible physiological resetting mechanism. These findings may serve as a catalyst for future investigation using prospective designs, multicenter data, and molecular approaches to elucidate the mechanisms underlying durable disease control.

## Conclusions

Our long-term surgical experience demonstrates that transsphenoidal surgery, when applied with refined patient selection and meticulous technique, offers excellent outcomes for women of childbearing age with enclosed-type prolactinomas. Notably, none of the women who conceived and delivered after surgical remission experienced recurrence, whereas non-pregnant women showed a high rate of recurrence. These findings suggest that achieving pregnancy after complete tumor resection may exert a protective effect and even provide the possibility of cure. We therefore propose a novel surgical indication for prolactinomas in women desiring pregnancy: achieving pregnancy under the assurance of complete tumor resection. This strategy not only enables early pregnancy planning and avoidance of lifelong medication but may also establish durable disease control. Further prospective multicenter studies and mechanistic investigations are warranted to validate this concept and elucidate the biological basis of sustained remission following surgery and pregnancy.

## Data Availability

The data that support the findings of this study are available from the corresponding author, [Amano K], upon reasonable request.
